# Diagnostic Precision of Anthropometric Variables for the Detection of Hypertension in Children and Adolescents

**DOI:** 10.3390/ijerph17124415

**Published:** 2020-06-19

**Authors:** Manuel Vaquero-Álvarez, Rafael Molina-Luque, Francisco Javier Fonseca-Pozo, Guillermo Molina-Recio, José López-Miranda, Manuel Romero-Saldaña

**Affiliations:** 1 Primary Care Emergency Service (SUAP) South Sanitary Area, 14850 Córdoba, Spain; manuel_vaquero1@hotmail.com; 2 Department of Nursing, Faculty of Medicine and Nursing, University of Córdoba, 14004 Córdoba, Spain; gmrsurf75@gmail.com (G.M.-R.); manuelromerosal@gmail.com (M.R.-S.); 3 Grupo Investigación GC12 Clinical and Epidemiological Research in Primary Care, Instituto Maimónides de Investigación Biomédica de Córdoba (IMIBIC), Hospital Universitario Reina Sofía, 14004 Córdoba, Spain; fjfonsecapozo@yahoo.es; 4 Department of Internal Medicine, Maimonides Biomedical Research Institute of Cordoba (IMIBIC), Reina Sofia University Hospital, University of Cordoba, 14004 Córdoba, Spain; jlopezmir@gmail.com

**Keywords:** anthropometry, high blood pressure, school population

## Abstract

*Introduction*: High blood pressure (HBP) is a health problem the prevalence of which has increased in young populations. Overweight and obesity in early ages have been directly related to its development. Due to the impact of HBP, it is necessary to provide tools that facilitate its early diagnosis, with useful anthropometric variables being those that assess obesity. The objective of this paper was to determine the diagnostic accuracy of anthropometric variables to detect HBP. *Methods:* A cross-sectional study was conducted on 265 students aged 6–16. The diagnosis of HBP was made following the criteria proposed by the Spanish Association of Pediatrics. Through different statistical methods, the association between anthropometric variables of general obesity with HBP was analyzed. *Results:* Waist circumference (WC) showed the best diagnostic capacity (area under the receiver operating characteristic curve = 0.729), with a sensitivity and specificity of 72.2% and 76%, respectively, for a cut-off point of 73.5 cm. In the adjusted multivariate analysis, an association was found between HBP and anthropometric variables: WC (odds ratio (OR) = 10.7), body mass index (OR = 7.5), waist-to-height ratio (OR = 5.5) and body fat percentage (OR = 5.3) (*p* < 0.05). *Conclusions:* The anthropometric variables studied showed a moderate predictive capacity for HBP, highlighting WC, which showed the strongest association with HBP in the infant and child population.

## 1. Introduction

Chronic non-communicable diseases (NCDs) are the leading cause of death worldwide, with cardiovascular diseases (CVDs) causing the largest number of deaths (17.8 million in 2017) [1]. They also represent a public health problem for countries due to their impact on the quality of life of people and their high economic burden [1,2,3].

The development of NCDs is linked to cardiovascular risk factors (CRF), where overweight and obesity stand out due to their high presence in our context. The overweight population has continuously increased in recent years, reaching a prevalence of obesity of 12% among adults worldwide by 2015 [4,5]. A worrying fact is the high proportion of children and young people who are overweight or obese. In Europe, it is estimated that 23.2% of children aged between 2 and 7 years are living with one or another disorder [6]. Besides, the trend has been observed to be upward overall. Between 1999 and 2016, in children aged 2–13, the prevalence of overweight grew from 20.6% to 21.3% and obesity from 4.4% to 5.7% [7]. In Spain, the prevalence rises to over 30% in the population between 8 and 17 years old [8].

This fact has caused the development of pathologies that are typically found in older age groups [9]. In this regard, the proportion of the children and adolescents with high blood pressure or hypertension (HBP) has increased [10,11]. The development of HBP in early ages is associated with a higher probability of NCDs in adulthood [12]. It is one of the leading causes of premature mortality [13]. Early intervention might lower associated morbidity [14].

It seems necessary to have tools for its early detection in order to avoid associated co-morbidities. It has been shown that anthropometric variables that allow assessment of the body fat distribution, such as waist circumference (WC), waist-to-height ratio (WHtR) or body mass index (BMI), are useful in child and youth populations [15]. Other advantages of these variables are the ease of measurement, non-invasiveness and straightforward interpretation. 

This study aimed to determine the diagnostic accuracy of anthropometric variables for the detection of HBP in a Spanish pediatric population.

## 2. Methods

### 2.1. Design Population Sample

A cross-sectional study was carried out on children and adolescents who were studying in primary and secondary schools in Pedro Abad (Córdoba) during 2018. From a population of 2000 subjects, for an expected prevalence of HBP of 6.1% [16], a safety of 95%, a power of 80% and a precision of 3%, a minimum sample size of 210 students was calculated. The final sample was composed of 265 children and adolescents, selected at random and stratified by age and sex.

Primary and secondary school students aged between 6 and 16 years were included, and they provided informed consent signed by themselves, parents/legal representatives and the principal investigator, depending on the age of the student. Children with rare diseases or cardiac pathology were excluded. 

### 2.2. Study Variables and Measures

Blood pressure (outcome variable) was determined through systolic blood pressure (SBP) and diastolic blood pressure (DBP) readings in mmHg. The measurement was made three times, with a five-minute interval between measurements, using the average of the last two. The procedure was carried out following the recommendations of the European Society for Hypertension in Children and Adolescents [17]. A research-validated automatic sphygmomanometer, model Omron M6 comfort^®^ (Kyoto, Japan), was used for measurement, with blood pressure cuffs adapted to the brachial circumference of the participants. The classification was made following the criteria proposed by the Spanish Association of Paediatrics (AEP), which establishes the percentiles of SBP and DBP for sex, age and size: Normotension (<P90), pre-high blood pressure (pre-HBP) (≥P90 − <P95) and HBP (≥P95) [18]. Additionally, these criteria were dichotomized into No HBP (<P95) and HBP (≥P95).

The independent variables collected were age (years), sex (boy/girl), BMI (kg/m^2^), WC (cm), WHtR, body fat percentage (BF%) and fat-free mass (FFM, kg). Nutritional status was assessed according to the World Health Organization criteria for BMI: underweight (<−2SD), normal weight (>−2SD or <+1SD), overweight (>+1SD or <+2SD) and obesity (>+2SD) [19]. 

Anthropometric variables were measured following the recommendations of the Reference Manual for the Standardization of Anthropometric Measurements [20]. Bodyweight, BF% and FFM were collected using an Omron BF-511^®^ (Omron Healthcare, Hoofddorp, The Netherlands) bioimpedance meter. Height was measured using a SECA 213^®^ (Hamburg, Germany) stadiometer. The WC was taken at the midpoint between the lower edge of the last rib and the highest point of the iliac crest at the end of inspiration and using a flexible stainless-steel tape measure. All the above measurements were measured with an accuracy of 0.1.

### 2.3. Ethical and Legal Aspects

The research protocol respected the principles of the Declaration of Helsinki, the World Medical Association and the Council of Europe Convention on Human Rights and Biomedicine. Inclusion in the study required the acceptance of the minor and the signature of informed consent by the legal representative. The protocol was also approved by the Bioethics Committee of Córdoba (Reference: 2353).

### 2.4. Statistical Analysis

The quantitative variables were presented with their mean and standard deviation and the qualitative variables with their absolute frequency and percentage. For the contrast of bivariate hypotheses, we used, when necessary, Student’s t-test, Analysis of Variance (ANOVA), Mann–Whitney U and Kruskal–Wallis tests.

The diagnostic accuracy of the independent variables for detecting HBP was evaluated using receiver operating characteristic (ROC) curves. The area under the curve (AUC), the cut-off point, the sensitivity, the specificity, the predictive values and the validity index of the main predictor variables were calculated through the Youden index (best sensitivity and specificity combined). Different calculations were used for the multivariate analysis:

- Adjusted multiple linear regression using SBP and DBP as the result variable. For determining the goodness-of-fit of the models, the standard error, the adjusted coefficient of determination, the F statistic, the collinearity analysis and the normality of the residues were analyzed.
- Multiple binary logistic regression using the HBP dichotomized (HBP yes/no) as outcome variable. Two models were developed, one using the independent variables in their continuous quantitative version, and the other using the dichotomized variables according to the best cut-off point obtained in the ROC curves. Adjusted odds ratio (OR) values with 95% confidence intervals were determined. Goodness-of-fit of the models was calculated through Cox-Snell’s R^2^, Nagelkerke’s R^2^, Hosmer-Lemeshow test and 2 log-likelihood.
- Discriminant analysis models adjusted only for quantitative predictive variables. Coefficients were obtained for each linear Fisher discriminant function (with or without HBP). The Box M test was used to contrast the equality of the matrices for the two groups (HBP or Not HBP), and the Wilks Lambda test was used to contrast the discriminant capacity before the predictive variables.

For all statistical analyses, an alpha error probability of less than 5% was accepted and the 95% confidence interval was calculated. Data analysis was performed using the software programs IBM SPSS Statistics v.22.0 (IBM, Chicago, IL, USA) and EPIDAT v.4.2 (Department of Sanidade, Xunta de Galicia, Galicia, Spain).

## 3. Result

### 3.1. Description of the Sample

Of the total 265 school children, 54.3% (95% CI 48.1–60.4%) were boys. The minimum and maximum ages were 6 and 17 years, respectively. The mean age of the sample was 11.2 years (95% CI 10.9–11.6 years), with 11.6 years (95% CI 11.1–12.1 years) for boys and 10.9 years (95% CI 10.5–11.3 years) for girls (*p* = 0.051).

The overall prevalence of HBP was 6.8% (95% CI 4.1–10.5%). A proportion of 6.3% (95% CI 2.9–11.5%) was found among boys, and 7.5% (95% CI 3.5–13.7%) among girls (*p* = 0.702). 

The overall prevalence of obesity was 23% (95% CI 18.1–28.6%), being higher in boys (25% (95% CI 18.2–32.9%)) than in girls (20.8% (95% CI 13.8–29%)), although without significant differences. Table 1 shows the main characteristics of the study sample. 

### 3.2. Diagnostic Accuracy of Anthropometric Variables for HBP

Table 2 shows the discriminant capacity of anthropometric variables for the diagnosis of HBP. The WC and BMI stand out with an AUC of 72.9% (95% CI 58.7–87.1%) and 71.8% (95% CI 58.3–85.3%), respectively. The FFM obtained the lowest AUC (47.8% 95% CI 33.5–61.9%), so its use as a predictor variable of HBP was excluded.

On the other hand, WC and BMI obtained the highest diagnostic accuracy (Table 2), with a Youden Index (JI) of 0.48 and 0.46, respectively. The WC for a cut-off point of 73.5 cm showed a sensitivity of 72.2%, specificity of 76% and validity index of 75.7%. In the case of BMI, the sensitivity, specificity and validity indexes obtained were 66.7%, 78.9% and 78%, respectively, with a cut-off point of 23 kg/m^2^. The negative predictive value of all variables studied was above 97%.

### 3.3. Predictive Multivariate Models for HBP

Table 3 shows the characteristics of the sample according to the outcome variable, which allowed us to identify those that could provide relevant information to build predictive models of HBP in children. Initially, all variables except sex and FFM showed significant differences between the group of healthy children and those with hypertension.

In order to determine the diagnostic accuracy of each of the anthropometric variables independently, different HBP predictive models were calculated (Table 4). 

SBP and DBP were used independently in multiple linear regression modelling. In the case of SBP, the variable that obtained the best goodness-of-fit (R^2^ = 0.467) was WC, followed by BMI (R^2^ = 0.430). DBP, WC, WHtR and BMI showed similar goodness-of-fit. However, their explanatory capacity (proportion of the dependent variable variation that can be explained by these independent variables) was low (R^2^ = 0.171, R^2^ = 0.153 and R^2^ = 0.137).

In the development of binary logistic regression models, anthropometric variables were used in their quantitative and dichotomous versions. The dichotomization (high/low) was performed according to the cut-off value shown in Table 2. In both versions, the WC was the one with the highest goodness-of-fit (R^2^ = 0.150 and R^2^ = 0.183, respectively), followed by BMI (R^2^ = 0.117 and R^2^ = 0.148).

The discriminant analysis showed that the WC was the only variable significantly associated with HBP, reaching 72.2% sensitivity. The coefficients of the discriminant function for HBP and normal blood pressure were WC = 0.621 and constant = −24.64, and WC = 0.532 and constant = −18.21, respectively.

## 4. Discussion

The aim of this paper was to determine the diagnostic accuracy of anthropometric variables for the detection of HBP in an infant/juvenile population aged between 6 and 16 years. 

The prevalence of HBP found was 6.8%, with no statistically significant differences between boys (6.3%) and girls (7.5%). A total of 21.6% of schoolchildren were pre-HBP. Although the proportion of children with HBP is higher than that observed by some authors, the results shown in other studies are not homogeneous, either nationally or internationally. There is great variability in the prevalence of pre-HBP, of HBP and in the differences observed between both sexes [7,21,22]. In our case, HBP was diagnosed using the oscillometric method, later confirmed with the auscultation method, to adapt to European recommendations [17].

This increasing prevalence of HBP has been associated with overweight and obesity, derived from the accumulation of fatty tissue [23]. For this reason, several authors have studied the predictive capacity (evaluated through AUC and sensitivity, specificity or predictive values) of anthropometric indexes that quantify the distribution of global and abdominal adiposity (WC, WHtR, BMI, BF%). In this regard, our results have shown heterogeneity in the association between the variables analyzed and HBP, somewhat in line with recent publications [15,24].

Among all the variables analyzed, WC showed a more robust association with HBP, regardless of the statistical method used, followed by BMI. WC is the parameter with the highest AUC (0.729). For a cut-off point of 73.5 cm, it reached a sensitivity, specificity and Youden index of 72.2%, 76% and 0.48, respectively. The BMI, at its cut-off point of 23 kg/m^2^, showed a lower Youden index (0.46) derived from a lower sensitivity (66.7%) and higher specificity (78.9%). This similar predictive ability is consistent with evidence from other works. Yamei et al. found that the BMI showed an AUC of 0.68 and the WC of 0.66, for HBP discrimination [24]. Furthermore, Christofaro et al. found no significant differences between the predictive ability of the two variables (WC_AUC_ = 0.59, BMI_AUC_ = 0.60; *p* = 0. 063) [25]. Some longitudinal studies have shown that both variables measured in childhood are equally able to predict the development of hypertension in adulthood, something that other parameters such as WHtR and waist-to-hip ratio do not achieve [26].

The results have shown that the increase in BMI (OR = 7.5 and OR = 1.19) and WC (OR = 10.7 and OR = 1.07) was more closely associated with HBP than other variables studied, an effect that other studies have shown [25,27]. The strength of this association is highly dependent on the population studied. The age range of the sample, the region or geographical area, and even ethnic differences seem to have an impact on the results obtained [25,28]. There are also variations in the existing criteria for assessing different cardiovascular risks, something which also affects HBP. This fact may lead to different results depending on the criteria used [29].

The relationship between anthropometric variables and SBP and DBP was also studied. For SBP, the parameters studied showed the goodness-of-fit greater than 0.4, with WC (R^2^ = 0.467) and BMI (R^2^ = 0.43). Once again, these variables show the best results. In the case of DBP, none of the variables had more than 20% goodness-of-fit. This trend, in which anthropometric measurements correlate better with SBP than with DBP, has been clearly shown by several studies [30,31,32,33]. Ortíz-Pinto et al. found that general and central obesity at four years was significantly associated with increased SBP and DBP at six years. This association was also observed among those who developed general or central obesity during the period of study [15].

The WHtR is gaining an important role because of its high predictive capacity for HBP risk, especially in adults [34]. In children and adolescents, there is no consensus regarding its predictive capacity [25,26,35]. In our population, although it showed a sensitivity over 70% and 0.428 goodness-of-fit for SBP, it was lower than those mentioned above. The main advantage of the WHtR is that it relates the central distribution of adiposity with respect to height (an especially important aspect in children of growing age). However, while other variables have an internationally accepted cut-off point (WC or BMI), this is not the case with the WHtR, which in some cases makes it difficult to assess [28,35].

Considering all the multivariate methods used, central obesity, measured through the WC, was best associated with the development of HBP. It should be added that it was the only significant variable in the discriminant analysis, reaching a sensitivity and specificity of over 70%.

In general, the predictive capacity of the variables is moderate, both in our results and those of other studies, which implies the need to develop more complex and effective models [24]. The difficulty of establishing optimal cut-off points for different populations and ages, due to the different characteristics of them, should be considered [35]. Some differences were observed even in populations with similar characteristics, which makes standardization more difficult [30].

Central adiposity plays a fundamental role in the pathogenesis of HBP [36]. The BMI or WC reduction over time has been associated with a decrease in the risk of developing HBP [15], which implies that early detection of high values of central or general adiposity should be considered, in order to perform early interventions to improve the health status of children and adolescents. These are simple variables to measure in any clinical setting, cost-efficient and have shown predictive capacity in different formats (percentiles, z-score, etc.) [32,37]. Furthermore, other variables that could help in early detection, such as wrist circumference or skin folds, should be studied further [27,35]. These new investigations could increase the number of tools available to aid when deciding which ones to use, depending on available resources.

Given the importance of HBP screening for early detection, the importance of including the assessment of these measures in the clinical routine has been highlighted [33], especially because blood pressure is not routinely measured in consultations [28,31,33]. This strategy would be beneficial for detecting risk even in those children who present with abdominal adiposity, regardless of their weight [38]. Increase in WC has been associated with the development of other CRFs [30].

## 5. Limitations

The study has various strengths and limitations. Although the sample size was kept to the minimum calculated, a larger number of children from various cities would ensure greater validity and applicability of the results. It should be note that the sample of pediatric students drawn from the Córdoba region of Spain may not be representative of the pediatric population in general.

## 6. Conclusions

Our study has shown that anthropometric indices for assessing general and central adiposity are closely associated with HBP in both female and male child and adolescent populations. Specifically, the WC is the variable that has shown the highest diagnostic accuracy and predictive capacity for the development of HBP, achieving the best results in different multivariate analyses, which highlights the importance of including the measurement of WC in the physical examination of children and adolescents in different clinical settings and even educational centers.

## Figures and Tables

**Table 1:** Sample characteristics.

Variables	Total (*n* = 265)	Boys (*n* = 144)	Girls (*n* = 121)	*p*
	Mean or N (SD or %)	Mean or N (SD or %)	Mean or N (SD or %)	
Age (years)	11.2 (2.9)	11.6 (2.9)	10.9 (2.9)	0.051
BMI (kg/m^2^)	20.5 (4.6)	20.7 (4.5)	20.2 (4.7)	NS
Underweight	2 (0.8)	1 (0.7)	1 (0.8)	NS
Normal weight	134 (50.8)	73 (50.7)	61 (50.8)	NS
Overweight	67 (25.4)	34 (23.6)	33 (27.5)	NS
Obesity	61 (23)	36 (25)	25 (20.8)	NS
Extreme Obesity	15 (5.7)	11 (7.6)	4 (3.3)	NS
WC (cm)	66.6 (11.5)	68.8 (12)	64.1 (10.3)	<0.01
WHtR	0.45 (0.06)	0.46 (0.07)	0.45 (0.05)	NS
BF%	24.4 (8.6)	23.6 (8.7)	26.3 (8.3)	<0.01
FFM (kg)	32 (4.4)	33.5 (4.8)	30.2 (3)	<0.001
SBP (mmHg)	111.4 (11.3)	112.8 (11.3)	109.6 (11)	<0.05
DBP (mmHg)	68.5 (6.3)	68.1 (5.9)	69 (6.7)	NS
Normal BP	189 (71.6)	103 (71.5)	86 (71.6)	NS
Pre-Hypertension	57 (21.6)	32 (22.2)	25 (20.8)	NS
High Blood Pressure	18 (6.8)	9 (6.3)	9 (7.5)	NS

BMI: body mass index; WC: waist circumference; WHtR: waist to height ratio; BF: body fat percentage; FFM: fat free mass; SBP: systolic blood pressure; DBP: diastolic blood pressure; BP: blood pressure.

**Table 2:** Area under curve for independent variables.

Performance	BMI	BF%	WC	WHtR	FFM
AUC (95% CI)	0.718 (0.583–0.853)	0.661 (0.545–0.776)	0.729 (0.587–0.871)	0.706 (0.593–0.819)	0.478 (0.335–0.619)
S.E.	0.069	0.059	0.071	0.059	0.072
*p*	<0.01	<0.05	<0.01	<0.05	0.75
Cut-off point	23	25.05	73.5	0.455	
Sensitivity (95% CI)	66.7 (42.1–91.2)	82.4 (61.3–100)	72.2 (48.8–95.7)	72.2 (48.8–95.7)	
Specificity (95% CI)	78.9 (73.6–84.2)	54.7 (48.3–61.1)	76 (70.5–81.6)	64.6 (58.8–70.8)	
PPV (95% CI)	18.8 (8.4–29.1)	11.2 (5.3–17.1)	18.1 (8.5–27.6)	13 (5.9–20.1)	
NPV (95% CI)	97 (94.4–99.6)	97.8 (95–100)	97.4 (94.9–99.9)	97 (94–99.9)	
Validity index (95% CI)	78.03 (72.9–83.2)	56.5 (50.3–52.7)	75.7 (70.4–81.1)	65.2 (59.2–71.1)	
Youden Index (95% CI)	0.46 (0.23–0.68)	0.37 (0.18–0.56)	0.48 (0.27–0.70)	0.37 (0.15–0.58)	

AUC: area under curve; S.E.: standard error; BMI: body mass index; WC: waist circumference; WHtR: waist to height ratio; BF: body fat percentage; FFM: fat free mass; PPV: positive predictive value; NPV: negative predictive value.

**Table 3:** Characteristics of the sample according to HBP.

Variables	Presence of HBP	Absence of HBP	*p*
Age (years)	12.7 (3)	11.1 (2.9)	<0.01
Sex			
Boys	9 (6.3%)	135 (93.8%)	NS
Girls	9 (7.5%)	111 (92.5%)	
BMI (kg/m^2^)	22.2 (5)	19.8 (4)	<0.001
≥23	12 (18.8%)	52 (81.2%)	<0.001
<23	6 (3%)	194 (97%)	
BF%	27.7 (9.2)	23.8 (8.1)	<0.05
≥25	14 (11.2%)	111 (88.8%)	<0.01
<25	3 (2.2%)	134 (97.8%)	
FFM (kg)	31.6 (4.6)	32.8 (4.3)	NS
WC (cm)	71.1 (13.4)	64.9 (10.1)	<0.001
≥73.5	13 (18.1%)	59 (81.9%)	<0.001
<73.5	5 (2.6%)	187 (97.4%)	
WHtR	0.48 (0.07)	0.45 (0.06)	<0.01
≥0.46	13 (13%)	87 (87%)	<0.01
<0.46	5 (3%)	159 (97%)	

BMI: body mass index; WC: waist circumference; WHtR: waist to height ratio; BF: body fat percentage; FFM: fat free mass; HBP: high blood pressure.

**Table 4:** Predictive models for high blood pressure.

**Multiple Linear Regression Adjusted by Sex and Age**				
**Dependent Variable**	**Independent Variable**	**B (S.E.)**	***p***	**R^2^**
SBP (mmHg)	BMI	0.598 (0.12)	<0.001	0.43
	WC	0.334 (0.05)	<0.001	0.467
	BF%	0.2 (0.06)	<0.01	0.392
	WHtR	41.3 (8.5)	<0.001	0.428
DBP (mmHg)	BMI	0.21 (0.009)	<0.05	0.137
	WC	0.15 (0.04)	<0.05	0.171
	BF%	0.08 (0.004)	<0.01	0.122
	WHtR	19.9 (6.04)	<0.01	0.153
**Logistic Regression Adjusted by Sex and Age (Categorical and Quantitative Variables)**				
**Dependent Variable**	**Independent Variable**	**OR Adjusted (95% CI)**	***p***	**R^2^ Nagelkerke**
HBP (Yes/No)	BMI *	7.5 (2.7–20.8)	<0.001	0.148
	WC *	10.7 (3.4–33.5)	<0.001	0.183
	BF% *	5.3 (1.49–19.1)	<0.05	0.122
	WHtR *	5.5 (1.9–16.3)	<0.05	0.145
HBP (Yes/No)	BMI	1.19 (1.08–1.32)	<0.001	0.117
	WC	1.09 (1.04–1.13)	<0.001	0.15
	BF%	1.06 (1.004–1.13)	<0.05	0.083
	WHtR	1.08 (1.03–1.26)	<0.001	0.115
**Discriminant Model Adjusted by sex, age, BMI, WHtR and BF%**				
**Dependent Variable**	**Independent Variable**	**Sensitivity**	**Specificity**	***p***
HBP (Yes/No)	WC	72.2%	72.4%	<0.001

* Qualitative variables; CI: confidence interval; HBP: high blood pressure; BMI: body mass index; WC: waist circumference; BF: body fat percentage; WHtR: waist to hight ratio; S.E.: standard Error; OR: odds ratio.
